# Editorial: Targeting metabolic disorders by trace elements and minerals: new insights and strategies

**DOI:** 10.3389/fnut.2025.1707942

**Published:** 2025-10-07

**Authors:** Hao-Long Zeng, Qing Yang, Di Wu

**Affiliations:** ^1^Department of Laboratory Medicine, Tongji Hospital, Tongji Medical College, Huazhong University of Science and Technology, Wuhan, China; ^2^Key Laboratory for Deep Processing of Major Grain and Oil, Ministry of Education, Wuhan Polytechnic University, Wuhan, China; ^3^Department of Chemistry, University of Oxford, Oxford, United Kingdom

**Keywords:** metabolic disorders (MD), nutrient metals, hypomagnesemia, nocturia, spontaneous preterm birth, metabolic syndrome, NAFLD

The intricate interplay between nutrition, metabolism, and human health is foundational to preventive and therapeutic medicine. Within this matrix, trace elements—whether essential or non-essential—operate as obligate cofactors in metabolic reactions and, when dysregulated, as pathogenic co-conspirators. The convergence of environmental changes, evolving dietary patterns, and the global burden of metabolic disorders necessitates a deeper understanding of how specific nutrients influence physiological pathways. The compelling studies by Dobrovolska and Boyarchuk, Su et al., Huang et al., Yu et al., and Hajhashemy et al., published in this Research Topic, provide a multi-faceted insight that significantly advances this field. Collectively, they underscore a central theme: the precise balance of nutrient metals is a potent, yet often overlooked, modulator of metabolic health across the lifespan, from prenatal development to adulthood, and across a spectrum of conditions from diabetes to liver disease.

Dobrovolska and Boyarchuk establish that children with T1DM display significant hypomagnesemia, even with dietary magnesium intakes comparable to controls. Serum magnesium levels decreased linearly with increasing HbA1c and neuroglycopenic symptoms, and with decreasing Ca^2+^/PO43− ratios. These findings position Mg2+ as a modifiable factor influencing both glycemic and neurological control in pediatric T1DM.

Expanding to young and middle-aged adults, Su et al. leveraged NHANES data to demonstrate a novel association between trace minerals and nocturia. Their cross-sectional analysis linked elevated cadmium and manganese with increased risk, while higher selenium was protective. This study implicates environmental toxicants and dietary minerals in urological health, suggesting mechanisms involving oxidative stress and hormonal disruption. A selenium threshold effect (< 2.15 μmol/L) offers a potential interventional target, arguing for the inclusion of trace mineral assessment in metabolic and urological health.

Huang et al. investigate prenatal cobalt's role in spontaneous preterm birth (SPB). They found a protective effect: moderate third-trimester maternal cobalt levels (0.63–1.07 ng/mL) significantly reduced SPB risk. Fasting blood glucose (FBG) mediated 9.12% of this association. This illustrates how a nutrient metal (cobalt) can influence obstetrical outcomes through metabolic homeostasis, challenging “more-is-better” paradigms and underscoring the concept of an optimal range.

The narrative is extended by meta-analyses from Yu et al. and Hajhashemy et al., which quantitatively synthesize the links between metals and metabolic disorders. Yu et al. establish a linear dose-response relationship between serum ferritin and the risk of metabolic syndrome (MetS) and non-alcoholic fatty liver disease (NAFLD). Each 50 μg/L ferritin increase was associated with a 15% (men) and 50% (women) higher MetS risk, and an 8% higher NAFLD risk. They note this association may be confounded by BMI and inflammation, highlighting the complex interplay between iron, adiposity, and inflammation. Hajhashemy et al. add complexity, revealing a definitive U-shaped relationship between blood selenium and MetS risk, with the lowest risk at 160 μg/L. This cautions against blanket selenium supplementation, underscoring the delicate balance between sufficiency and toxicity.

Together, these five studies form a powerful and nuanced narrative: the imbalance of nutrient metals—whether deficiency (magnesium), excess (cadmium, manganese, iron), toxicity (high selenium), or suboptimal levels (cobalt, selenium)—is a critical factor in the pathogenesis of diverse metabolic, hepatic, and inflammatory disorders across all ages and organ systems. These findings offer novel therapeutic avenues for metabolic disorders, including: (1) Routine screening of specific nutrient metals in at-risk populations; (2) Personalized nutrition plans, with mineral intake tailored to an individual's health status, life stage, and baseline nutrient levels; (3) Public health strategies to improve dietary quality and access to nutrient-rich foods in vulnerable communities; (4) Therapeutic interventions, such as controlled iron depletion or selenium repletion, which represent potential strategies for managing MetS and NAFLD.

To advance these implications, further research is required. While epidemiological associations are clear, the underlying molecular mechanisms—such as magnesium's role in insulin signaling, selenium's anti-inflammatory effects, cobalt's influence on glucose metabolism, and iron overload's promotion of hepatic steatosis—require elucidation. Rigorous randomized controlled trials are needed to test if targeted supplementation or dietary modification improves clinical outcomes. Furthermore, future studies must adopt an exposome approach, investigating the mixture effects of simultaneous exposure to complex metal cocktails, as interactions between elements like iron, selenium, and inflammatory status may be pivotal for metabolic health.

In conclusion, the work presented here argues for a more sophisticated view of nutrient metals that moves beyond the simplistic lens of deficiency syndromes. It demonstrates that their role is dichotomous, acting as either essential factors or toxicants based on precise concentration, individual context, and complex physiological interactions. Understanding this duality is critical for developing novel, targeted strategies to prevent and manage the global burden of metabolic disorders.

